# An Enigmatic Soft-Tissue Creeping Phenomenon: The Spontaneous Peri-Implant Mucosa Margin and Papilla Growth, Part Two—A Scientifically Supported Hypothesis Article

**DOI:** 10.3390/dj12070216

**Published:** 2024-07-12

**Authors:** Ivo Agabiti, Karol Alí Apaza Alccayhuaman, Zenzaburo Taniguchi, Kazuhisa Kuwano, Daniele Botticelli

**Affiliations:** 1 ARDEC Academy, 47923 Rimini, Italy; agabitiivo@gmail.com; 2 Department of Oral Biology, University Clinic of Dentistry, Medical University of Vienna, 1090 Vienna, Austria; caroline7_k@hotmail.com; 3 Department of Oral Implantology, Osaka Dental University, 8-1 Kuzuhahanazonocho, Hirakata 573-1121, Japan; zenshin_base@yahoo.co.jp (Z.T.); kuwano_kazuhisa@yahoo.co.jp (K.K.)

**Keywords:** implant, mucosa growth, peri-implant mucosa, keratinized tissue, crown overcontour, BOPT, papilla growth

## Abstract

In our previous article, we observed and measured a spontaneous growth in the coronal direction of the keratinized tissues present around implants. This growth involved both free margins and interdental papillae, and we indicated our hypothesis on the probable cause of this still-unexplained phenomenon. The growth of oral soft tissues involves several other structures, such as the linea alba and tongue indentation. Our idea holds that growth of these tissues is generated by the negative intraoral pressure created in the oral phase of swallowing and the subsequent resting position, which through the resulting suction causes a shift of these soft structures in the gaps around the dental crowns. Other hypotheses have been suggested in the past to understand this phenomenon of soft tissue growth, which still lacks data supporting etiological evidence. The purpose of this article is to thoroughly analyze and verify our model by comparing the clinical observations with citations and examples from the literature, combined with notions of physiology, biology, and physics that help in clarifying these events. To better explain the mechanisms of oral soft tissue growth, photographs of clinical cases paradigmatic of the phenomenon are shown.

## 1. Introduction

In dental prosthetic restorations, poor esthetic outcome is often brought about by missing interdental papillae that appear as black triangles and exaggerated lengthening of clinical crowns due to recession of the marginal soft tissues. Reconstruction of these lost anatomical structures often requires attempting invasive surgical methods such as gingival grafts or complex plastic surgery flaps.

It has been observed in our previous work that gingival papillae and margins can spontaneously grow in the coronal direction [1]. Various conditions have been described as necessary for the phenomenon to occur: the presence of a band of crestal keratinized mucosa adequate in width and thickness [1]; the presence and location of the contact point [2,3]; a congruous distance between teeth or implants [4,5]; an optimal bucco–lingual width of the alveolar ridge (buccal–lingual thickness of the crest) [6], and finally, the presence of empty spaces around the prostheses that can be filled by crestal soft tissues [1,7].

However, all the conditions just listed do not explain the mechanism of papilla and marginal soft tissue growth around crowns. For papilla growth, an inflammatory hypothesis was presented by Jemt in 1997 [8]. He stated that inflammatory soft tissues increase in volume and tend to spontaneously grow and fill interdental gaps. He also proposed his own papillary index by defining five levels of filling, from “no papilla”, value 0, to “hyperplastic papilla”, value 5. It seems obvious that inflammation leads to an increase in tissue volumes. However, this inflammation was not detected in our previous report [1].

A more likely hypothesis as a cause of papillary growth, in agreement with our thinking, concerns the pump effect on soft tissue due to the negative intraoral pressure produced during swallowing [1,7]. For about 150 years, many authors have described the formation of negative pressure during the oral phase of swallowing [9,10,11,12,13,14,15,16,17,18,19,20].

Engelke described two oral “functional sucking spaces” in which a negative pressure was measured during swallowing [21]. These “bio-functional compartments” are composed of a sub-palatal compartment (sucking space; Donders 1875) [9] and an interocclusal space (interocclusal negative pressure space; Fränkel 1967) [15]. The entity of negative pressure is greater in the sub-palatal than in the interocclusal compartment. This negative pressure is maintained with a plateau of lesser value until the seal of the lips is reopened, restoring atmospheric pressure [13,22].

Engelke [21] examined on lateral radiographs of the skull the change in the position of the tongue on the palate by comparing radiograms before and after voluntary swallowing and confirmed the radiographic observations made by Eckert-Möbius [13], namely the lifting and adhesion of the lingual surface against the hard and soft palate after swallowing.

In fact, the formation of the velo-lingual seal after swallowing contributes to the stabilization of the tongue in the oral cavity, which is self-maintained due to negative pressure, without the need for muscular actions during this resting position of the tongue [15,21]. This could mean that it is the negative pressure that sucks the tongue onto the palate and not the muscle action of pushing the tongue against the palate [9].

Negative pressure draws the tongue to the sub-palatal and interocclusal spaces, which also includes the interdental spaces. This negative pressure must be measured with manometer or pressure sensors capable of measuring negative values. If we use pressure sensors designed specifically to measure positive values and place them between the tongue and palate, they will naturally register a positive pressure.

Fränkel’s interocclusal space is a functional compartment that encompasses and interconnects the interocclusal and interdental spaces between the teeth, allowing for air and/or fluid communication along the entire dental arch [21]. This compartment is bounded buccally by the soft tissues of the cheeks/lips and orally by the tongue. When the tongue is lowered, Fränkel’s space can transiently connect with the space beneath the palatine vault.

Engelke clinically demonstrated the formation of negative pressure in Fränkel’s interocclusal space during and after voluntary swallowing [21]. By employing a device connected to the oral cavity, he visualized a membrane depression during swallowing with the mouth closed, indicating the presence of a negative pressure inside the oral cavity.

If the mouth is completely sealed (both posteriorly and anteriorly), the negative pressure generated during swallowing can be maintained in the interocclusal and sub-palatal spaces for longer periods, albeit at reduced levels [19]. When swallowing, the lower dental arch makes contact with the upper arch. However, after swallowing, the mandible returns to its resting position, increasing the inner volume of the closed mouth and consequently creating a greater intraoral negative pressure. This negative pressure persists until the lips are closed, albeit at lower values. This means that the pump effect [1,7] produced during swallowing may persist over time. In our previous work [1], implant-supported prostheses were over-contoured at the apical buccal third of the prosthetic crown to create a collar zone, simulating a false root. This allowed us to create an empty volume not accessible by cheeks, lips, and tongue in which the negative pressure could act as a pump, sucking the keratinized crestal tissues to fill the embrasures and empty spaces at the collar of the crowns. The same phenomenon might induce soft tissue formation, filling over time any empty, inaccessible oral space. This might also explain the formation of linea alba, tongue indentation, and other soft tissue growths.

The purpose of this article is to thoroughly analyze and verify our model by comparing the clinical observations with citations and examples from the literature, combined with notions of physiology, biology, and physics that help clarifying these events. To better explain the mechanisms of oral soft tissue growth, photographs of clinical cases paradigmatic of the phenomenon are shown. For more information, please refer to our previously published work [1] [link for the open-access article].

## 2. Clinical Examples of Oral Soft Tissue Growth

**Figure 1:**
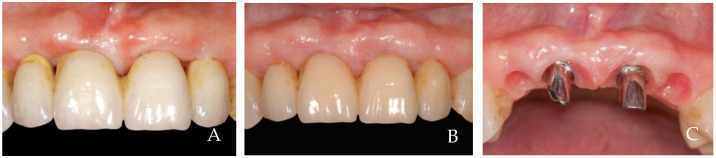
A 63-year-old female patient, non-smoker, who does not use drugs that induce gingival hyperplasia and does not exhibit bruxism. (**A**) Implant prosthesis at the time of delivery. Note the ischemia of the compressed tissues and the lack of papillae. (**B**) Follow-up at 6 years. The papillae have formed, and the tissue margins have migrated coronally, covering the narrowest part at the collar of the crowns which simulated the roots. (**C**) Note the growth of the tissues and papillae after prosthesis removal.

**Figure 2:**
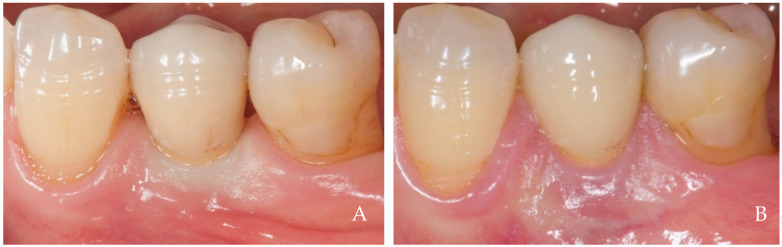
A 61-year-old female patient, non-smoker, who does not use drugs that induce gingival hyperplasia and does not exhibit bruxism. (**A**) Crown supported by an implant in position 3.4 at the time of delivery. (**B**) Two-year follow up. Note the growth of papillae that included the neighboring natural teeth. A minimal recession was observed buccally at position 3.4 probably due to the over dimensions of the false root and to the reduced thickness of the alveolar crest, conditions that did not allow the creation of a horizontal recessed zone.

**Figure 3:**
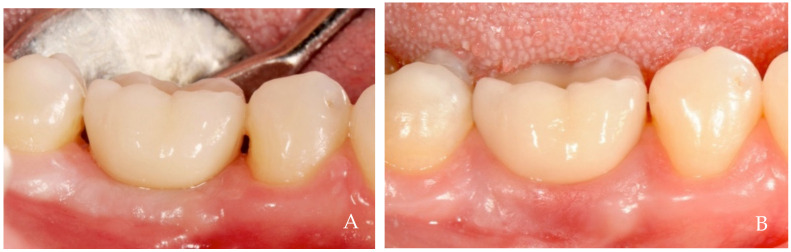
A 25-year-old female patient, non-smoker, who does not use drugs that induce gingival hyperplasia and does not exhibit bruxism. (**A**) Crown supported by implant in position 4.6 at the time of delivery. Note the absence of the two papillae and the ischemia at the collar due to tissue compression. (**B**) Six-month follow up. Note the coronal growth of the peri-implant mucosa and of the papillae that included the neighboring natural teeth. The lack of contact point probably prevented the complete filling of the mesial papilla, maybe because of the lack of a hermetical sealing of this embrasure.

**Figure 4:**
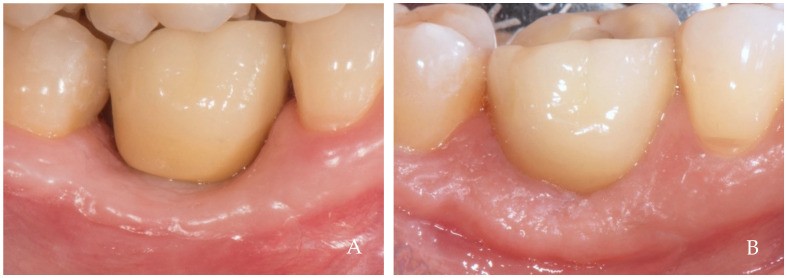
A 34-year-old female patient, non-smoker, who does not use drugs that induce gingival hyperplasia and does not exhibit bruxism. (**A**) Crown supported by implant in position 4.6 at the time of delivery. (**B**) Four-year follow-up. Observe the complete filling of the papillae and the coronal growth of the peri-implant keratinized tissues.

**Figure 5:**
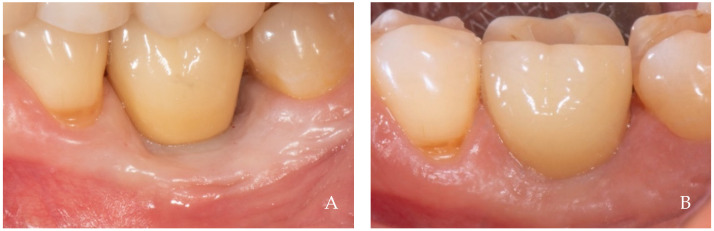
A 34-year-old female patient, non-smoker, who does not use drugs that induce gingival hyperplasia and does not exhibit bruxism. (**A**) Crown supported by implant in position 3.6 at the time of delivery in the same patient in Figure 4. (**B**) Four-year follow-up. The coronal growth of the peri-implant keratinized tissues was incomplete on the distal aspect, likely due to the mesial inclination of tooth 3.7, which created a papillary embrasure that was too wide to establish a negative pressure sufficient for optimal papillary growth.

The keratinized tissue surrounding the crowns grows over time spontaneously, filling the empty interdental spaces and therefore favoring the formation of the papillae (Figure 1, Figure 2, Figure 3, Figure 4 and Figure 5) [1]. As we have already assumed, the intraoral negative pressure produced during the oral phase of swallowing can influence the growth of keratinized tissues around the prostheses. To enable this tissue growth, certain conditions already described are necessary, such as the distance between implants and/or roots (Figure 5) and the position of the contact point (Figure 3). Among these conditions, one of the most important is the anatomy of the crown. If the prosthetic crowns are built with an over-contoured profile in the apical third, they are necessarily determined recessed zones around the collar, creating local anatomies similar to (or even exaggerated) [23] the enamel–cement junction (Figure 1, Figure 2, Figure 3, Figure 4 and Figure 5). In this case, the lips, cheeks, and tongue lean over the most protruding parts of the crowns and of the alveolar ridges, establishing an underlying empty chamber around the collar, already defined as an inaccessible area by Morris [23] and subsequently defined an inaccessible space by Agabiti [1].

Negative pressure attracts every intraoral soft tissue toward the empty spaces indifferently. Mew affirmed that continuous and light muscular forces produce a major effect on the bones of the cranium compared to heavy and alternating forces [24]. The heavy and alternating forces are determined by muscular contractions, while continuous and light forces are mainly postural as in resting conditions. The former condition is present during swallowing, while the latter is present during the rest position after swallowing, when the lips are closed and normal nose breathing occurs. In the rest position, with the lowering of the mandible, the intraoral space is augmented so that a light and negative pressure is maintained, creating a continuous sucking of soft tissues. This suction mechanism might be responsible for soft tissue growth [1,7].

The keratinized tissues can grow and maintain their form and position easily due to their firm attachment to the bone crest. Furthermore, these tissues are thick and tough, rich in dense connective tissue, which helps to maintain structures such as the papillae more stably overtime.

When the conditions necessary for tissue growth are met, keratinized tissues occupy the inaccessible spaces. However, when these conditions are not met, such as when the recessed spaces increase in dimension, physical factors (Bernoulli’s law) decrease the effectiveness of the negative pressure, thereby hindering the growth of keratinized crestal tissues [1,7].

**Figure 6:**
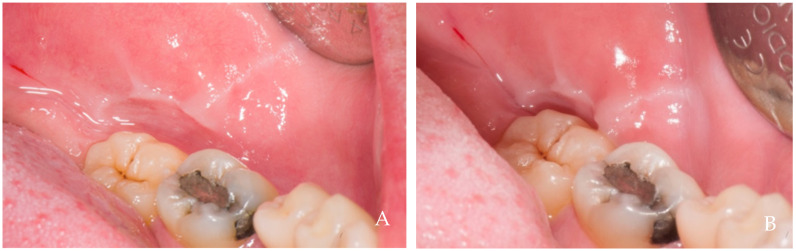
A 36-year-old female patient, non-smoker, who does not use drugs that induce gingival hyperplasia and does not exhibit bruxism. (**A**,**B**) The linea alba is a thickened fold of tissue on the cheek corresponding to the occlusal plane, often attributed to mechanical trauma from clenching. However, this explanation fails to account for the vertical formations visible in this clinical case, which align with the interdental spaces of the upper jaw. Alternatively, all linea alba formations may result from negative pressure that can draw the cheeks against the teeth, leading to the adaptation of soft tissues to the dental crowns, resembling an impression.

**Figure 7:**
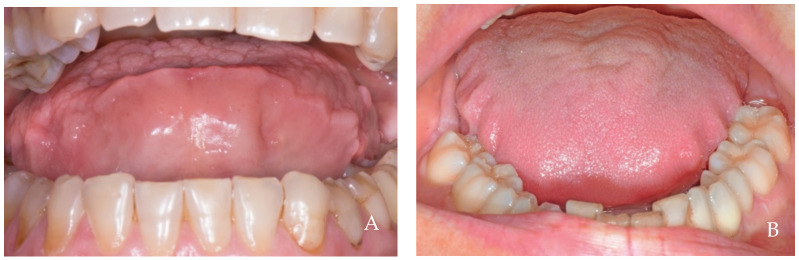
(**A**) A 58-year-old female patient, non-smoker, who does not use drugs that induce gingival hyperplasia and exhibits bruxism. (**B**) A 39-year-old female patient, non-smoker, who does not use drugs that induce gingival hyperplasia and does not exhibit bruxism. Two examples of lingual indentations that likely share the same formation mechanism as the linea alba, as these conditions are often associated. Negative intraoral pressure can pull the tongue tissue against the teeth, filling the interocclusal spaces when the arches are separated, as well as the interdental spaces, for instance, in the mandibular rest position. This mechanism differs significantly from the lingual muscle thrust described by some authors.

**Figure 8:**
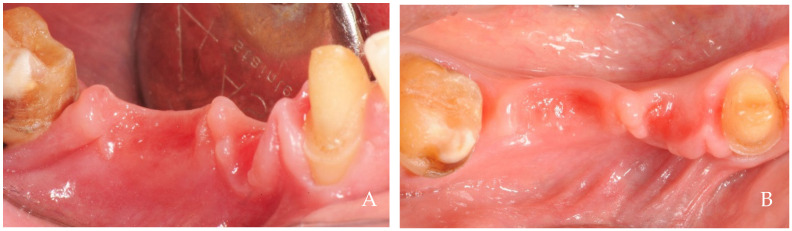
(**A**,**B**) A 62-year-old female patient, non-smoker, who does not use drugs that induce gingival hyperplasia and does not exhibit bruxism. Another case of keratinized tissue growth under an old bridge that had de-cemented. Note the increase in marginal soft tissue only in the keratinized area, while in the area of the alveolar mucosa, the phenomenon did not occur due to the reasons reported in the text.

**Figure 9:**
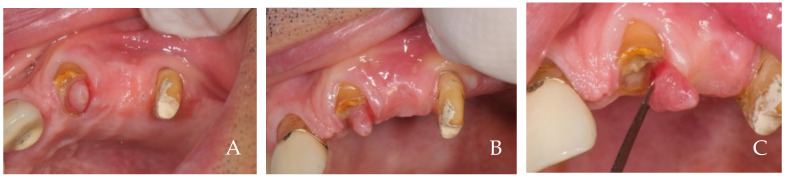
(**A**–**C**) A 65-year-old female patient, smoker, who does not use drugs that induce gingival hyperplasia and does not exhibit bruxism. During the removal of an old bridge, hyperplastic keratinized tissue was discovered inside the crown of the bridge, resulting from the total erosion of the tooth due to decay, which was gradually replaced by soft tissues. It can be speculated that the negative pressure in this area may have created conditions conducive to tissue growth.

**Figure 10:**
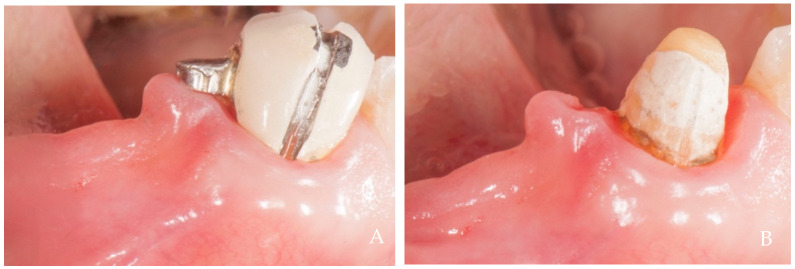
A 65-year-old female patient, non-smoker, who does not use drugs that induce gingival hyperplasia and does not exhibit bruxism. Keratinized tissues formed beneath the attachment of a removable prosthesis both (**A**) before and (**B**) after the removal of the prosthetic crown. Intraoral negative pressure might have led to the suction of soft tissues beneath the space containing the attachment, resulting in the formation of a papilla-like keratinized tissue.

**Figure 11:**
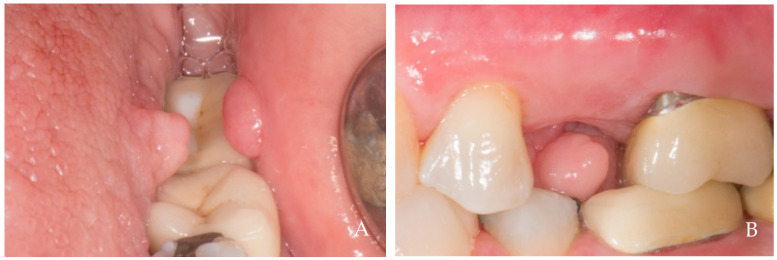
(**A**,**B**) 36-year-old female patient, non-smoker, who does not use drugs that induce gingival hyperplasia and does not exhibit bruxism. Newly formed tissue has grown in mirror-like positions on the tongue and cheeks, corresponding to the empty space left by the missing tooth 2.5. Intraoral negative pressure may have caused the soft tissues to be suctioned into the empty space.

**Figure 12:**
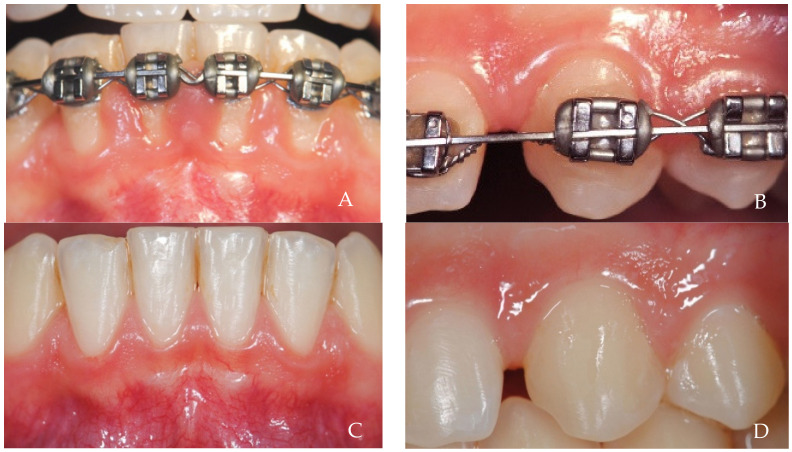
A 26-year-old female patient, non-smoker, who does not use drugs that induce gingival hyperplasia and does not exhibit bruxism. The challenges of maintaining good oral health while wearing orthodontic brackets are well known. However, inflammation alone cannot be solely responsible for such exaggerated papilla growth, and negative pressure may also have an important effect. (**A**,**B**) depict situations before bracket removal in the same patient. (**C**,**D**) Six-month follow-up.

On the other hand, the alveolar mucosa is a thin, non-keratinized tissue that undergoes continuous movement during chewing, speaking, swallowing, and other oral functions, resulting in a constant change in its shape, making it impossible to maintain a stable form [25,26]. Nevertheless, in other conditions, the tissues of the lips, cheeks, and including those of the tongue may also undergo hyperplastic activities. Among them, we can identify structures that protrude beyond their natural anatomical profile, such as the linea alba structures (Figure 6), tongue indentations (Figure 7), marginal keratinized tissue growth (Figure 8), empty spaces filled by hyperplastic keratinized tissue (Figure 9 and Figure 10), hyperplastic formations on the tongue and cheeks (Figure 11), and hyperplastic papillae around fixed orthodontic braces (Figure 12). Gingival hyperplasia during orthodontic treatment was evaluated in 193 patients [27]. After logistic regression analysis, only two factors were significantly associated with gingival enlargement: metallic brackets and the duration of treatment. No positive correlation was found with plaque amount, indicating that other cause–effect factors must be involved.

## 3. Discussion

Some authors attribute the linea alba formations to clenching and bruxism [28,29,30,31,32], while other authors did not find any association [33]. However, in another study, no variations in pressure on the teeth exerted by the buccal mucosa were observed, except during swallowing [34]. During swallowing, the buccinator muscle shows no electrical activity, indicating it does not contribute to pushing the mucosa against the teeth. However, anatomically, buccal mucosa ridging corresponds to the area of the buccinator muscle, while the masseter muscle, which is involved in swallowing, is located more posteriorly and externally. This suggests that the function of the masseter muscle cannot exert pressure on the teeth at the site of mucosa ridging [34]. Nevertheless, the association between buccal mucosa ridging and clenching/bruxism reported in some studies [31,32] allowed us to hypothesize that the higher biting force in bruxers might induce higher negative pressure at least during swallowing and that this pressure is maintained in the rest position until the lips are opened.

Instead, sucking habits or the physiological process of swallowing might be responsible for the formation of mucosa ridging [34,35]. Additionally, it should be considered that a basic, slight negative pressure exists even at rest [36], which can continuously draw the cheek mucosa into the interocclusal and interdental spaces.

Regarding lingual indentations, some studies suggest they might result from clenching and bruxism [29,30,37]. However, a clinical study involving 244 adults found no statistically significant correlation between mucosa ridging and tongue indentations towards awareness of grinding and clenching [35]. This suggests that similar assumptions about intraoral negative pressure might apply to tongue indentations as well. It indicates that the formation of indentations might be attributed to suction of the tongue towards the interdental spaces rather than an active thrust of the tongue against the teeth.

In our opinion, there is a basic misunderstanding. Many studies have measured only negative tongue pressures, while others have found only positive or both positive and negative pressures. This depends on the aim of the research and of the instrument used for measurements.

In the absence of muscle activity, other mechanisms must be involved. The negative pressure seems to be the most plausible mechanism to draw newly formed soft tissues within inaccessible spaces to the tongue, cheeks, and lips.

Despite several authors having reported the presence of an intraoral negative pressure, few authors have considered the effect of negative pressure on the growth of oral soft tissues within empty (inaccessible) spaces. It is important to acknowledge that parameters such as distance from the bone crest to the contact point, distance between implants or teeth, presence of keratinized tissues, crown contouring, and others are critical for papilla growth. However, while these factors are necessary conditions, they cannot be considered causal factors. On the contrary, negative pressure appears to play a significant role, suggesting it as a potential causal factor in papilla growth. This concept must be considered when designing the shape of prosthetic elements to ensure optimal aesthetics. Poor design can lead to unsatisfactory aesthetic results, compromising the overall outcome. It is advisable to leave inaccessible spaces [23] in areas where tissue growth is desired, adhering to principles such as the presence of keratinized mucosa, the distance of the contact point from the bone crest, the distance between elements, and all other factors that might be involved in the process of soft tissue growth.

The limitations of hypothesis articles are numerous. In the present study, it is important to note that the conclusions are based on speculative arguments without experimental data that clearly demonstrate the causal effect of negative pressure on tissue growth. The tissue growth around prostheses supported by implants was shown in photographs and reported in a retrospective study, which also has its own limitations [1]. Speculative hypotheses can lead to misleading or incorrect conclusions and may suggest interventions without sufficient evidence.

Nevertheless, this article attempts to provide a hypothesis framed within a well-supported scientific basis. However, without clear evidence of a causal effect, caution must be exercised when considering any intervention in patients. It should also be noted that several variables must be considered for spontaneous tissue growth around prostheses supported by implants. Among these variables, the most important are the presence of keratinized mucosa, the distance between elements, the level of the contact point, the buccal–lingual papilla width, the thickness of the mucosa (biotype), the presence of inaccessible spaces (embrasures between elements, overcontour of the crown), the type of neighboring elements, and the level of the papilla on these neighboring elements. However, while these factors are necessary conditions, none of them can be considered a causal effect for tissue growth. There is the theory that inflammation caused by plaque can induce hyperplasia, but this cannot be considered healthy tissue growth. Some drugs may also induce hyperplasia, but this would also occur in other locations in the periodontal or peri-implant mucosae. Moreover, considering additional data on patients included in the previously published work [1], none of the 61 patients included were taking drugs that could induce hyperplasia, and only 6 patients were smoking more than 10 cigarettes per day. No inflammation was detected in any of the 103 sites evaluated that could induce edema or evident inflammatory infiltrate. No soft tissue grafts were performed. Papilla growth occurred in sites where inaccessible spaces [23] were intentionally created, and in most cases, tissue growth was also observed buccally.

## 4. Conclusions

Many factors influencing soft tissue growth are necessary conditions that facilitate tissue development but do not establish a direct cause-and-effect relationship. Among these potential factors, biofilm accumulation and inflammatory processes, which include edema and inflammatory reactions, were not observed in the present study. Drug-induced hyperplasia was also not applicable, as none of the patients were taking such medications. This study demonstrated that soft tissue growth occurred precisely in areas where inaccessible spaces were either intentionally created or naturally present. This suggests that additional factors must be involved, with the sucking effect generated by intraoral negative pressure being a plausible candidate. However, conclusive research demonstrating a causal relationship between negative oral pressure and keratinized tissue growth is still lacking.

## Data Availability

The present hypothesis article does not contain data.
